# Investigating the effect of multimedia education in combination with teach-back method on quality of life and cardiac anxiety in patients with heart failure: a randomized clinical trial

**DOI:** 10.1186/s12872-021-02357-z

**Published:** 2021-11-12

**Authors:** Fateme Mohammadi, Mitra Sadeghi Jahromi, Mostafa Bijani, Shanaz Karimi, Azizallah Dehghan

**Affiliations:** 1 Chronic Diseases (Home Care) Research Center and Autism Spectrum Disorders Research Center, Department of Nursing, Hamadan University of Medical Sciences, Hamadan, Iran; 2 Department of Medical Surgical Nursing, Fasa University of Medical Sciences, 81936-13119 Fasa, Iran; 3 NonCommunicable Diseases Research Center (NCDRC), Fasa University of Medical Sciences, Fasa, Iran

**Keywords:** Anxiety, Education, Multimedia, Heart failure, Quality of life, Teach-Back

## Abstract

**Background:**

Education can contribute to promotion of the quality of life and reduction of heart anxiety in patients with heart failure, so it is important to find a suitable educational method for these patients. Therefore, the present study was an attempt to determine the effect of multimedia education using teach-back method on the life quality and cardiac anxiety in patients with heart failure.

**Methods:**

The present study was a randomized clinical trial. 120 patients with heart failure class I to III and aged less than 60 years old were selected using sequential sampling; then, they were assigned randomly into two intervention groups and one control group. Group A (multimedia education), group B (education using multimedia together with teach-back method), and group C (control). The quality of life and cardiac anxiety were evaluated in the participants of the three groups before, after, 1 month, and 3 months after the intervention. Data were analyzed using descriptive tests, Pearson correlation, Kolmogorov–Smirnov, chi square and ANOVA test in SPSS 22. The significance level was set at *P* < 0.05.

**Results:**

No significant differences were found in the mean scores of the quality of life and cardiac anxiety in the control and two intervention groups before the educational intervention. However, immediately after, 1 month and 3 months after the educational intervention, a significant difference was observed between the mean scores of the quality of life and cardiac anxiety in the intervention groups (*P* < 0.05).

**Conclusion:**

Multimedia education together with Teach-Back method is effective in promoting the quality of life and reducing cardiac anxiety in patients with heart failure. Therefore, it is recommended that health policymakers should use this educational method in providing treatment programs.

***Iranian Registry of Clinical Trials*:**

20190917044802N1.

Registration date: 5/2/2020.

## Introduction

Heart failure is one of the major challenges in the healthcare systems of all countries [1]. The American Heart Association has reported that heart disease is responsible for 22% of deaths every year [2, 3]. In Iran, heart failure is the most common cause of hospitalization because about 3,700 per 100,000 Iranians suffer from heart failure [4]. However, impaired functional ability is one of the most important consequences of this disease, and patients experience limitations in occupational, familial, and social responsibilities, which consequently affect the quality of life of these patients [5]. Therefore, due to the chronic, progressive, and irreversible nature of this disease, decreasing the anxiety and improving the quality of life in these patients are among the most important responsibilities of the healthcare systems [6]. In this regard, holding educational interventions is one of the most important measures taken to improve awareness and subsequently reduce anxiety and improve the quality of life of these patients [6, 7]. Although many studies in the last decade have examined the impact of education on the quality of life of patients with heart failure, they have used traditional educational methods such as lectures, educational booklets, and pamphlets. [8]. A study has suggested that up to 40–80% of medical information is immediately forgotten and much of the information recalled is inaccurately retained [9]. Therefore, in recent years, due to being client-centered, new educational approaches have received much attention in patient education [10].

In this regard, multimedia and teach-back education methods have been emphasized because of their important role in active learning in patients [11]. Multimedia education, as a new educational method makes the transfer of the content easier, deeper and more attractive through the use of images, videos and audio, consequently leading to better learning than traditional methods in learners [12]. In addition, an intervention that has been shown to be effective on improving information retention and understanding is the teach-back method [13]. The Teach Back/Show Back Method is a teaching technique [14], In this method, the instructor teaches the client in a simple and understandable language, using no specific medical terms, and the client is then asked to retell his/her understanding in his/her own language. Thereafter, if the client did not understand the materials well, the instructor repeats them until the client fully understands them. Accordingly, this process allows the nurses to evaluate the patient's understanding and the accuracy or otherwise of his/her information [14, 15].

On the other hand, educating patients is one of the most important tasks of nurses to reduce anxiety, improve self-care, and promote the quality of life of these patients [16–18]. Anxiety disorders in patients with heart failure are common and associated with adverse outcomes such as reduced adherence to treatment, poor function, frequent hospitalization, and decreased quality of life [19]. It is necessary for the treatment team, especially nurses, to play an important role through educational interventions in reducing anxiety and improving the quality of life in patients with heart failure [20]. Although the scientific texts mostly emphasize using new methods in educating patients, examination of some scientific literature showed that few studies have used a combination of methods in Iran and other countries. Most of the educational materials for patients in hospitals and health centers in Iran are based on educational pamphlets. Therefore, the present study aimed to examine the effect of multimedia education together with teach-back method on the quality of life and cardiac anxiety in patients with heart failure in the south of Iran, in 2020.

## Methods

The present study is a non-blinded, randomized controlled study conducted in one of the specialized heart hospitals in the south of Iran from April to October 2020. Because of the obvious nature of the intervention, patients and field researchers could not be blinded. Data collection and analysis was conducted by a neutral researcher who was not involved in data acquisition. Design of the study was recorded at the clinical trials center (IRCT20190917044802N1).

In the current study, the inclusion criteria were patients with heart failure (class I to III) aged less than 60 years old (confirmed by the cardiologists based on their echocardiographic findings and New York Heart Association (NYHA) Functional Classification); patients with no speech, vision, and hearing impairments; literacy and ability to talk and answer questions. Further, the exclusion criteria were patients who were absent in more than two sessions; those with mental illnesses such as Alzheimer’s disease, acute cerebrovascular diseases, or chronic illnesses such as Alcoholic liver disease (ALD) (i.e. cirrhosis), cancer, rheumatoid arthritis, or acute heart failure; and patients who were not able to communicate. In the present study, CONSORT (Consolidated Standards of Reporting Trials) checklist was used for determining the quality of randomized controlled trials [21]. The sample size for this study was calculated based on Abbasi et al.’s study with a power of 80% and α = 0.05, using a formula [22]. Therefore, about 37 patients were decided to be enrolled in each group, but given the loss of about 10% of the samples during the study, the sample size was finally considered 40 people in each group.

$$n = \frac{{\left( {Z_{{1 - \frac{\alpha }{2}}} + Z_{1 - \beta } } \right)^{2} \left( {\delta_{1}^{2} + \delta_{2}^{2} } \right)}}{{\left( {\mu_{1} - \mu_{2} } \right)^{2} }}$$

**Fig. 1:**
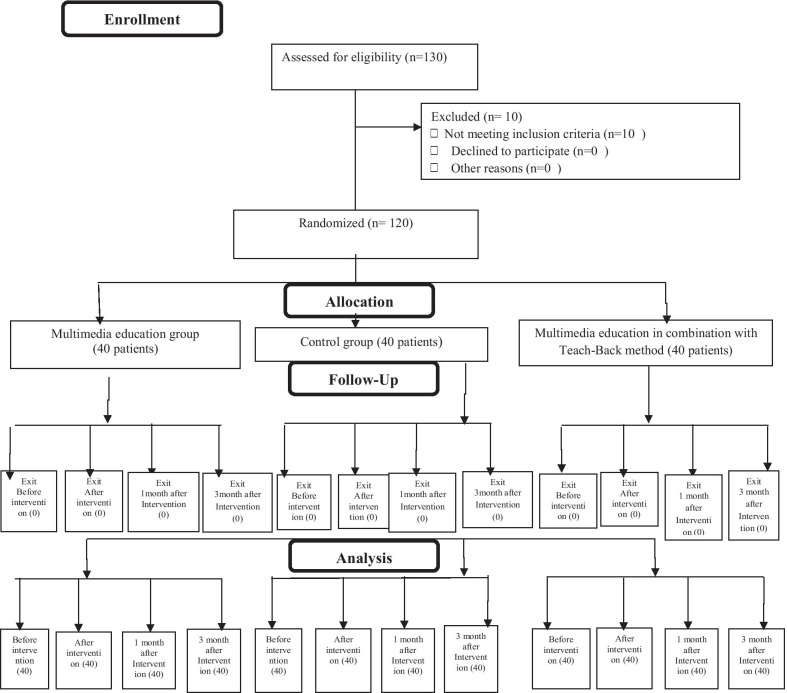
CONSORT Flow diagram of the project

The first author invited and registered 130 patients to participate in the study by sequential sampling. Of them, 10 patients who were reluctant to participate in the study or did not meet the inclusion criteria were excluded. Therefore, the remaining 120 patients were then allocated randomly to the three groups by the second author, including a control group, intervention group A (multimedia education), and intervention group B (multimedia education together with teach-back method). Thereafter, 120 cards, including 40 cards labeled A (intervention group with multimedia education), 40 cards labeled B (intervention group with multimedia education together with Teach-Back method), and 40 cards labeled C (control group), were prepared. These 120 cards were then put in an envelope and each patient was asked to draw out one card randomly. Each card labeled A, B, and C, were the two intervention groups and one control group. Figure 1 presents the consort flow diagram of the participants throughout the study (Fig. 1). At the beginning of the study, the researcher explained about the objectives of the educational program and emphasized the importance of the participants’ punctuality to achieve better results at the end. The corresponding author was responsible for teaching and leading the group discussion in the both intervention groups.

The films (educational CDs) were specific multimedia used for the intervention group (the intervention group A); they were screened in four sessions, each one lasting for 45 min. The content of the educational films was prepared in consultation with a cardiologist, a nutritionist, a psychologist, and a number of nursing professors. Of note, the films were produced in one of the virtual education centers of Fasa University of Medical Sciences in the south of Iran. In this regard, the content of these educational CDs included the signs and symptoms of heart failure, methods of prevention of heart complications, methods of drug consumption and diet regimen in patients with heart failure, and relaxation techniques used in order to control anxiety and live a happier life.

In the intervention group B, teach-back method was used after the film screening. Accordingly, both film screening and teach-back learning performed in four 75-min sessions in this group. Thus, the patients were asked to reflect on what they had watched in the films; then, they were requested to reflect on their experiences and express them. These sessions continued in the form of group discussions, questions and answers, responses to the patients' ambiguities and questions, and feedback to them. Finally, the patients were asked to talk about their care plans in the future. In this method, education continued until the participants fully understood the contents of the educational films, and if any patient had any questions, he/she was provided with the needed explanation in simple language. For example, the patients were asked to talk about their diet plans based on what they have learned from the educational films and explanations.

In the control group (group C), the educational program was performed according to the hospital's daily routines such as the ward nurses’ education and the educational package presentation.

To collection the data, we used the demographic questionnaire (age, sex, marital status, level of education, comorbidities, level of heart failure), Minnesota living with heart failure questionnaire (MLHFQ), and Cardiac Anxiety Questionnaire (CAQ). Finally, the research assistant, who was not aware of the allocation of participants in the groups, distributed and gathered the questionnaires before, after (immediately), 1 month, and 3 months after the intervention.

## Data collection tools

### Minnesota living with heart failure questionnaire (MLHFQ)

This questionnaire, which was designed by Rector, is one of the mostly used tools which determine the health-related quality of life of patients with heart failure. Accordingly, it assesses the patients' understanding and perceptions of the effects of heart failure (HF) on both physical and psychological aspects of their lives. It includes 21 questions and two subscales. The physical domain has 8 items, including questions 7, 6, 2, 4, 12, 5, 13, and 3; the emotional domain has 5 items, including questions 17, 18, 19, 20, and 21. The remaining 8 items are calculated in the total score. Each item is scored in a 6-point Likert Scale ranging from 0 (no impairment) to 5 (very much impairment). The total score is calculated by the sum of the scores of all these items, which ranges from 0 to 105. The lower the total scores of this questionnaire, the better the quality of life of patients with heart failure. The reliability of this questionnaire was previously confirmed with a Cronbach’s alpha coefficient of 0.89 [23]. Eskandari et al. [24] in their study translated this questionnaire into Persian and examined its content validity and reliability. They estimated a reliability of 0.90 for this questionnaire.

### Cardiac anxiety questionnaire (CAQ)

This questionnaire was firstly developed by Eifert et al. to assess cardiac anxiety. It includes 18 questions and 3 subscales, consisting of fear domains (items 10, 11, 13, 14, 15, 16, 17, and 18), avoidance domain (items 2, 5, 7, and 12), and heart-focused attention domain (items 1 3, 4, 6, and 8). Each item is scored in a 5-point Likert Scale ranging from 0 (never) to 4 (always). The higher scores indicate higher level of the patient’s anxiety. The reliability of this questionnaire was confirmed with a Cronbach’s alpha coefficient of 0.87 [25]. Raiesdana et al. in their research translated this questionnaire into Persian and confirmed the reliability of this questionnaire with Cronbach’s alpha coefficient of 0.89 [26].

### Data analysis

Data analyses were conducted using SPSS version 22. To analyze the data, we used descriptive statistics (namely frequency, percentage, mean, standard deviation). Kolmogorov–Smirnov test showed that the data were normally distributed. Thus, inferential statistics applied included Chi-square and ANOVA test (comparison of data distribution among groups), ANOVA test (comparison of the mean scores of the quality of life and cardiac anxiety among groups), and repeated measure test (intra-group comparison of the mean scores of the quality of life and cardiac anxiety). *P* values less than 0.05 were considered statistically significant for all tests.

### Ethical considerations

All participants signed the informed consent to participate in the study. The present study was conducted in accordance with the principles of the revised Declaration of Helsinki, a statement of ethical principles which directs the physicians and other participants in medical research involving human subjects. The participants were assured of their anonymity and confidentiality of their information Moreover, the study was approved by the local Ethics Committee of Fasa University of Medical Sciences (Ethical code: IR.FUMS.REC.1398.099).

## Results

Out of 120 patients included in this study, 80 (66.7%) were men and 40 (33.3%) were women. The patients’ age ranged was 20–60 years, with a mean of 49.54 ± 2.74 years old. There was no significant difference in demographic variables among the intervention and control groups (Table 1).

**Table 1:** Demographic characteristics of the patients

Variables	Total	Control group	Multimedia education group	multimedia education in combination with Teach-Back method	*P *value
*Gender*					
Male	80 (66.7%)	25 (62.5%)	27 (67.5%)	28 (70%)	0.769*
Female	40 (33.3%)	15 (37.5%)	13 (32.5%)	12 (13%)	
*Age groups*					
Under 45	37 (80.8%)	14 (35%)	13 (32.5%)	10 (25%)	0.602*
Over 45	83 (69.2%)	26 (65%)	27 (67.5%)	30 (75%)	
*Marriage status*					
Single	8 (6.7%)	2 (5%)	3 (7.5%)	3 (7.5%)	0.875*
Married	117 (93.3%)	38 (95%)	37 (92.5%)	37 (92.5%)	
*Education level*					
Under diploma	43 (35.81%)	19 (47.5%)	12 (30%)	12 (30%)	0.113*
Diploma	27 (22.5%)	7 (17.5%)	12 (30%)	8 (20%)	
Associate's degree	12 (10%)	1 (2.5%)	7 (17.5%)	4 (10%)	
Bachelor's degree	38 (31.7%)	13 (32.5%)	9 (22.5%)	16 (40%)	
*Has diabetes*					
Yes	52 (42.3%)	15 (37.5%)	15 (37. %5)	22 (55%)	0.190*
No	68 (56.7%)	25 (62.5%)	25 (62.5%)	18 (45%)	
*Has hypertension*					
Yes	61 (50.8%)	19 (47.5%)	23 (57.5%)	19 (47.5%)	0.587*
No	59 949.2%)	21 (51.5%)	17 (42.5%)	21 (51.5%)	
*Other disease*					
Yes	104 (86.7%)	33 (82.5%)	37 (92.5%)	34 (85%)	0.538*
No	16 (13.3%)	7 (17.5%)	3 (7.5%)	6 (15%)	
*Physical activity*					
Yes	52 (42.3%)	18 (45%)	17 (42.5%)	17 (42.5%)	0.231*
No	68 (56.7%)	22 (55%)	23 (57.5%)	23 (57.5%)	
*Smoking*					
Yes	44 (36.7%)	15 (37.5%)	15 (37.5%)	14 (85%)	0.9650*
No	76 (63.3%)	25 (62.5%)	25 (62.5%)	26 (65%)	
*Days of hospitalization*					
Days > 3	12 (10%)	4 (10%)	5 (12.5%)	3 (7.5%)	0.825**
3–6 days	76 (81.7%)	30 (75%)	26 (65%)	30 (75%)	
Days < 6	22 (18.3%)	6 (15%)	9 (22.5%)	7 (17.5%)	
*Ejection fraction*					
> 20	4 (3.3%)	1 (2.5%)	2 (5%)	1 (2.5%)	0.638**
20–40	58 (48.3%)	23 (57.5%)	18 (45%)	17 (42.5%)	
41–60	58 (48.3%)	16 (40%)	20 (50%)	22 (55%)	

Values are expressed as no. (%)

*Chi-square test

**ANOVA test

Repeated Measures ANOVA showed no significant differences were also found in the mean scores of the quality of life and cardiac anxiety in the intervention and control groups before performing the educational intervention. However, a significant difference was observed in the mean score of quality of life and cardiac anxiety in the intervention and control groups immediately after, 1 month, and 3 months after the educational intervention (*P* < 0.05) (Tables 2, 3).

**Table 2:** Comparison of the quality of life at different time points among the groups

Group	Before intervention	After intervention	1 month after the intervention	3 month after the intervention	*P* value*
Control group	36.75 ± 15.17	39.55 ± 17.27	45.82 ± 13.10	45.72 ± 12.94	< 0.001
Multimedia education group	40.6 ± 14.73	30.25 ± 14.23	25.62 ± 10.44	35.32 ± 10.35	< 0.001
Multimedia education in combination with Teach-Back method	39.67 ± 13.45	18.37 ± 11.92	14.67 ± 9.05	13.32 ± 6.67	< 0.001
*P* value**	0.395	< 0.001	< 0.001	< 0.001	

*ANOVA test

**Repeated measure test

**Table 3:** Comparison of the cardiac anxiety at different time points among the groups

Group	Before intervention	After intervention	1 month after the intervention	3 month after the intervention	*P* value*
Control group	29.69 ± 9.63	31.25 ± 1.62	36.62 ± 9.08	36.67 ± 8.97	< 0.001
Multimedia education group	32.25 ± 10.86	21.15 ± 8.66	18.6 ± 7.04	18.47 ± 6.99	< 0.001
multimedia education in combination with Teach-Back method	31.32 ± 10.41	11.37 ± 6.95	9.37 ± 5.85	8.72 ± 5.74	< 0.001
*P* value**	0.590	< 0.001	< 0.001	< 0.001	

*ANOVA test

**Repeated measure test

The post hoc test (Tukey test) was used to examine the difference among the means. No significant differences were found in the mean scores of quality of life and cardiac anxiety in the intervention and control groups before the educational intervention. However, a significant difference was observed in the mean score of quality of life (*P* < 0.01) and cardiac anxiety (*P* < 0.001) in the intervention and control groups immediately after, 1 month, and 3 months after the intervention. Also, the findings of this study revealed the differences mean scores of quality of life and cardiac anxiety in multimedia education together with teach-back method was more than control groups and multimedia education group (Tables 4, 5).

**Table 4:** A comparison of the difference between the mean scores of quality of life in the intervention and control group using the Tukey test

Time	Control group		Multimedia education in combination with Teach-Back method		Multimedia education group	
	Mean ± SD	*P* value	Mean ± SD	*P* value	Mean ± SD	*P* value
Before—(immediately after)	2.8 ± 6.91	0.014	10.84 ± 5.96	< 0.001	21.30 ± 10.53	< 0.001
Before—1 month	9.7 ± 6.1	< 0.001	14.97 ± 6.20	< 0.001	25 ± 13.71	< 0.001
Before—3 months	8.97 ± 6.54	< 0.001	15.27 ± 6.52	< 0.001	26.35 ± 11.02	< 0.001

**Table 5:** A comparison of the difference between the mean scores of cardiac anxiety in the intervention and control group using the Tukey test

Time	Control group		Multimedia education in combination with Teach–Back method		Multimedia education group	
	Mean ± SD	*P* value	Mean ± SD	*P* value	Mean ± SD	*P* value
Before—(immediately after)	2.12 ± 4.14	0.003	11.10 ± 5.45	< 0.001	19.95 ± 9.45	< 0.001
Before—1 month	6.94 ± 5.41	< 0.001	13.65 ± 5.25	< 0.001	31.95 ± 7.90	< 0.001
Before—3 months	6.97 ± 5.42	< 0.001	13.77 ± 5.56	< 0.001	22.6 ± 8.14	< 0.001

## Discussion

Heart failure is a debilitating and deadly chronic disease that affects various aspects of patients' quality of life. It is also associated with high complications and death, as well as a significant amount of health care cost [27]. Patients with heart failure have a poor quality of life due to having insufficient knowledge on the acceptance and adherence to the correct treatment regimen and timely diagnosis and control of the related symptoms [28]. Patient’s education is the main key to have effective management and control of heart failure. If a timely diagnosis and a proper patient’s education for health care be established, one can prevent or delay the complications of heart failure and then promote patients’ quality of life [29].

The results of the present study indicated that both multimedia education and multimedia education together with teach-back method were effective in reducing cardiac anxiety and promoting quality of life in patients with heart failure compared to traditional methods. Also, it was found that multimedia education together with teach-back method was more effective than multimedia education alone in reducing cardiac anxiety and promoting quality of life of patients with heart failure.

In our study, it was found that multimedia education reduces cardiac anxiety and increases the quality of life of the heart patient one day, 1 and 3 months after the intervention. Consistent with this study, other studies have reported the effectiveness of multimedia education in reducing anxiety and increasing self-care and quality of life. In this regard, findings of the study carried out by Kola et al. supported the use of the multimedia approach increased the self-care in one day before and fore and eight weeks after discharge in patients with heart failure, and increasing self-care improves the quality of life in these patients [30]. Bakogiannis et al. also shows that the use of the multimedia educational of heart failure can increase quality of self-care and consequently the quality of life in these patients [31]. The study conducted by Abbasi et al. also showed that the multimedia educational method was effective on increasing the level of awareness and promoting self-care behaviors and the quality of life of patients with heart failure compared to traditional teaching methods [22].

Boyde et al. also stated that multimedia educational intervention can be effective in decrees cardiac anxiety and increase the level of health and quality in people with heart failure and consequently reducing their readmissions [32]. The above studies examined the multimedia educational approach had more positive effects on self-care behaviors and the quality of life in patients with heart failure. This similarity may be due to the educational approach and the patient population being similar. The study conducted by Torabizadeh et al. and Boyer et al. also showed that the multimedia educational caused a reduce anxiety in patients with heart diseases. This finding is consistent with the present study [33, 34].

In the present study, multimedia education together with teach-back method reduces cardiac anxiety and increases the quality of life of the heart patient one day, 1 and 3 months after the intervention. Rahmani et al. stated that teach-back method increased knowledge; performance and quality of life in heart failure patients immediately after teachback education and 3 months after discharge [35]. Also, study of Kola et al. show teach-back method increasing the level of knowledge led to promote of self-care behaviors, patient-caregiver self-reporting and quality of life in these patients [30]. In addition, Tran et al. showed that teach-back education caused a longer retention of the information learned and improved the effectiveness of health outcomes [36].

Contrary to the findings of present study, Ross et al. showed that the teach-back education was not statistically significant on quality of life scores in patients with heart failure. The reason for this difference can be due to the difference in sample size, sample chosen (stages of HF from NYHA Class I-IV), living conditions and financial considerations [37]. But in line with the present study, Kollia et al. and Klingbeil et al., showed that teach-back method caused a reduce anxiety in patients with heart diseases. This finding is consistent with the present study [27–38].

In this study, it was also revealed that multimedia education together with teach-back method compared to the multimedia educational method was more effective in reducing cardiac anxiety and increasing the quality of life. Consistent with this finding, Kola et al. state multimedia education together with teach-back training compared with multimedia educational can lead to a higher improvement in patients' knowledge, self-care behaviors and quality of life in patients with heart failure. Because increasing the patients' knowledge led to the promotion of self-care behaviors and quality of life [30]. In line with the present study, Srisuk et al. revealed that the multimedia training along with patient-caregiver collaboration can improve the knowledge levels in the caregivers and also self-care behaviors in patients with heart failure [39].

*The limitations of the study* This study did not examine some physiological indicators such as blood pressure (BP), heart rate (HR), ejection fraction (EF), and patients' knowledge about heart failure. It is recommended that these parameters should be examined in future studies.

*Strengths* This study was the first clinical trial which compared the effects of two new educational methods on the quality of life and cardiac anxiety in patients with heart failure, and this strength distinguishes this study from other studies performed in this field. Furthermore, some specific questionnaires were used to assess the quality of life and cardiac anxiety in patients with heart failure.

## Conclusion

Although both multimedia education and multimedia education together with Teach-Back education were found to be effective in promoting the quality of life and reducing cardiac anxiety in patients with heart failure, multimedia education together with reflective learning showed a better effectiveness than multimedia education in the long run, which can be due to the longer retention of the information learned using this method. Therefore, it is suggested that managers and policymakers of the health system should use this educational approach to promote the quality of life and self-care behaviors in patients with heart failure and other chronic diseases.

## Data Availability

The datasets used and/or analysed during the current study are available from the corresponding author on reasonable request.

## Abbreviations

HF: Heart failure
NYHA: New York Heart Association
SD: Standard deviation
